# Notes on the *Stenus cirrus* group, with description of two new species from China (Coleoptera, Staphylinidae)

**DOI:** 10.3897/zookeys.169.2647

**Published:** 2012-02-10

**Authors:** Yu-Hong Pan, Liang Tang, Li-Zhen Li

**Affiliations:** 1 Department of Biology, Shanghai Normal University, 100 Guilin Road, 1st Educational Building 323 Room, Shanghai, 200234 P. R. China

**Keywords:** Coleoptera, Staphylinidae, *Stenus cirrus* group, identification key, new species, China

## Abstract

Two new species, *Stenus zhangdinghengi* **sp. n.**, *Stenus maoershanus* **sp. n.**,of the *Stenus cirrus* group are described from South China, Guangxi Province. The male of *Stenus fellowesi* Puthz, 2003 and the female of *Stenus huanghaoi* Tang & Li, 2008 were discovered for the first time. Their diagnostic characters are illustrated and a key to the Chinese species of the *Stenus cirrus* group is provided.

## Introduction

The *Stenus cirrus* group is a large group of the genus with 57 species worldwide and 24 species in China. The members of the group are characterized by the presence of long and erect setae on the abdomen. A detailed group definition was given by Puthz (2009).

Among the specimens we collected from China recently, the male of *Stenus fellowesi* Puthz, 2003, described from Hainan Province, and the female of *Stenus huanghaoi* Tang & Li, 2008, described from Guangdong Province, were discovered for the first time. Two species of the *Stenus cirrus* group collected from Guangxi Province are recognized as new and described for the first time.

## Material and methods

The specimens examined in this paper were collected by sifting leaf litter in forests. For an examination of the male genitalia, the last three abdominal segments were detached from the body after softening in hot water. The aedeagi, together with other dissected parts, were mounted in Euparal (Chroma Gesellschaft Schmidt, Koengen, Germany) on plastic slides. Photos of sexual characters were taken with a Canon G9 camera attached to an Olympus SZX 16 stereoscope; habitus photos were taken with a Canon macro photo lens MP-E 65 mm attached to a Canon EOS40D camera.

The type specimens treated in this study are deposited in the following public and private collections:

SHNU Department of Biology, Shanghai Normal University, P. R. China

cPut private collection V. Puthz, Schlitz, Germany

cRou private collection G. de Rougemont, London, England

The measurements of proportions are abbreviated as follows:

BL body length, measured from the anterior margin of the clypeus to the posterior margin of abdominal tergite X

FL forebody length, measured from the anterior margin of the clypeus to the apicolateral angle of elytra

HW width of head including eyes

PW width of pronotum

EW width of elytra

PL length of pronotum

EL length of elytra, measured from humeral angle

SL length of elytral suture

## Taxonomy

### Stenus fellowesi

Puthz, 2003

http://species-id.net/wiki/Stenus_fellowesi

Figs 1–2 9–21

#### Material examined.

**CHINA: Hainan Prov.: Holotype:** ♀,Mt. Diaoluoshan, alt. 1040 m, 24.V.1999, J. R. Fellowes leg. (cRou). **Other material:** 42♂♂, 30♀♀, Mt. Diaoluoshan, Diaoluozhandao, alt. 930–1000 m, 20–23.IV.2010, YIN Zi-Wei, FENG Ting & YUAN Xiao-Zhuan leg. (1♂, 1♀ in cPut, remainder in SHNU).

Male. Sternite VII (Fig. 9) impressed in posteromedian portion with emargination along posterior margin of impression, impression densely setose; sternite VIII (Fig. 10) with a triangular emargination at middle of posterior margin, length of the emargination about 1/ 5 of total length along the midline; sternite IX (Fig. 11) with long and slightly acute apicolateral projections, posterior margin nearly straight; tergite X (Fig. 12) with posterior margin truncate. Median lobe of aedeagus (Fig. 13) broad near base and tapering apicad, apex of median lobe (Fig. 14) forming an acute projection with two pairs of short setae; parameres (Fig. 14) slightly longer than median lobe, swollen at apex, each with about 14 to 15 setae on apico-internal margins.

**Figures 1–2.**
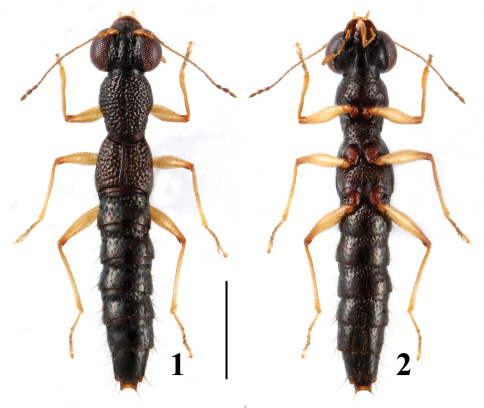
Habitus of *Stenus fellowesi* in dorsal and ventral view. Scale = 1 mm.

#### Variation.

The duct of the spermatheca may be folded to different degrees (Figs 18–21).

#### Distribution.

China (Hainan Province: Diaoluoshan).

### Stenus huanghaoi

Tang & Li, 2008

http://species-id.net/wiki/Stenus_huanghaoi

Figs 3–4 22–30

#### Material examined.

**CHINA: Guangdong Prov.: Holotype:** ♂, Ruyuan County, Nanling Nature Reserve, alt. 1019 m, 18.VI.2007, HUANG Hao & XU Wang leg. (SHNU). **Other material:** 1♂, Ruyuan County, Nanling Nature Reserve, alt. 1500–1800 m, 17.VIII.2008, QI Nan & YIN Zi-Wei leg. (SHNU); 2♀♀, Ruyuan County, Nanling Nature Reserve, alt. 1100 m, 14.VIII.2008, QI Nan & YIN Zi-Wei leg. (SHNU); 1♂, Shaoguan City, Nanling Nature Reserve, alt. 700 m, 18.VIII.2010, TANG Liang leg. (SHNU).

Female. Sternite VIII (Fig. 27) with posterior margin indistinctly prominent in the middle; tergite X (Fig. 28) with posterior margin broadly rounded and slightly emarginated at apex; valvifers (Fig. 29) each with big tooth at apex, posterior margin serrate; strongly sclerotized spermatheca very simple (Fig. 30).

**Figures 3–4.**
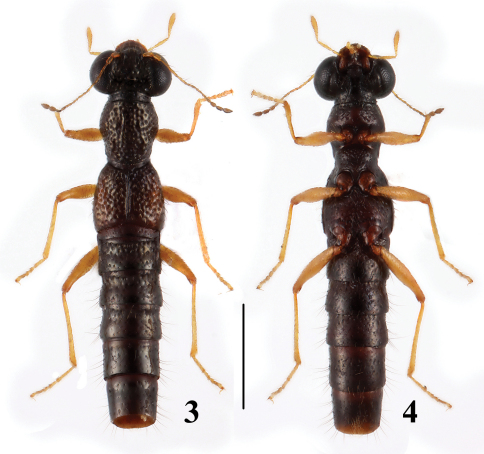
Habitus of*Stenus huanghaoi* in dorsal and ventral view. Scale = 1 mm.

#### Notes.

In the original description, the elytral mark of the species was described as obsolete. In fact, there is an indistinct elongate orange mark in the lateral portion of each elytron (Fig. 3).

#### Distribution.

China (Guangdong Province: Nanling).

### Stenus zhangdinghengi

Pan, Tang & Li sp. n.

urn:lsid:zoobank.org:act:01C1CD74-66DF-445E-BED7-3F6B00E5EEBE

http://species-id.net/wiki/Stenus_zhangdinghengi

Figs 5–6 31–40

#### Type material.

**Holotype:** **CHINA: Guangxi Prov.:** ♂, Lingui County, Huaping Nature Reserve, Anjiangping, alt. 1400–1700 m, 14.VII.2011, PENG Zhong leg. (SHNU). **Paratypes:** **CHINA: Guangxi Prov.:** 4♂♂, 7♀♀, Lingui County, Huaping Nature Reserve, Anjiangping, alt. 1300–1700 m, 14–18.VII.2011, TANG Liang , HE Wen-Jia & PENG Zhong leg. (1♂, 1♀ in cPut, remainder in SHNU).

#### Description.

 BL: 3.5–3.9 mm; FL: 1.6–1.8 mm.

HW: 0.72–0.79 mm, PW: 0.53–0.55 mm, PL: 0.55–0.60 mm, EW: 0.60–0.68 mm, EL: 0.59–0.66 mm, SL: 0.44–0.49 mm.

Brachypterous; body brownish except for the blackish head, anterior margin of labrum, antennae, maxillary palpi and legs yellowish brown, each elytron with an elongate ill-defined orange mark near lateral side, this mark 1/3 to 1/2 as long and about 1/3 to 2/5 as broad as the respective elytron.

Head 1.16–1.21 times as wide as elytra; interocular area with two broad longitudinal furrows, median portion convex dorsally, not reaching level of inner eye margins; punctation round and slightly confluent, uniform except for several large punctures at posterior part of median portion; diameter of large punctures about as wide as antennal segment II in cross-section, interstices between punctures smooth, mostly narrower than half the diameter of punctures, those along midline much wider, forming a broad impunctate line. Relative length of antennal segments from base to apex 12: 9: 20: 11: 10.5: 10.5: 9: 6.5: 7: 8: 10.5. Paraglossae oval.

Pronotum 1.05–1.09 times as long as wide, 0.81–0.88 times as wide as elytra; disk with shallow median longitudinal furrow about 1/2 the length of pronotum; punctures round and moderately confluent, smaller in size than largest punctures on head, interstices smooth, narrower than half the diameter of punctures.

Elytra 0.96–0.98 times as long as wide, distinctly constricted at base, lateral margins gently divergent posteriad; disk almost even; punctures round to elliptic, uniform, slightly coarser than those of pronotum, interstices smooth, narrower than half the diameter of punctures.

Legs with hind tarsi 0.72–0.78 times as long as hind tibiae, tarsomere IV strongly bilobed.

Abdomen cylindrical; paratergites very narrow and smooth, present only at abdominal segment III, tergite VII with indistinct palisade fringe; punctation of tergite III–VIII sparse and shallow, gradually becoming finer posteriad, interstices smooth, varying from narrower to much wider than diameter of punctures.

Male. Sternite VII with shallow emargination at middle of posterior margin and a depression before it; sternite VIII (Fig. 31) with semi-circular emargination at middle of posterior margin; sternite IX (Fig. 32) with long apicolateral projections, posterior margin serrate; tergite X (Fig. 33) with posterior margin convex. Aedeagus (Fig. 34) with apical sclerotized portion of median lobe pointed at apex; expulsion hooks (Fig. 36) very large; parameres longer than median lobe, slightly swollen at apex, each with about 14 short setae at apico-internal margins.

Female. Sternite VIII (Fig. 37) with posterior margin entire; tergite X (Fig. 38) with posterior margin convex. Valvifers (Fig. 39) each with large apicolateral tooth; sclerotized spermatheca as in Fig. 40.

**Figures 5–6.**
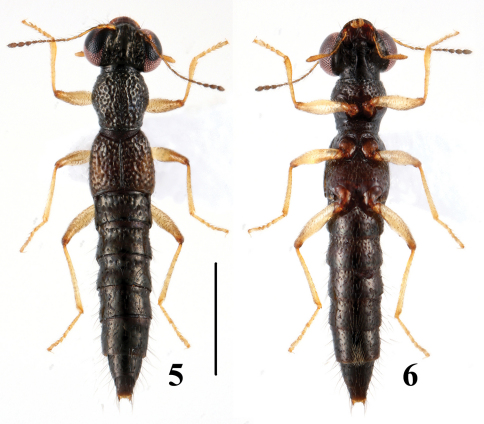
Habitus of *Stenus zhangdinghengi* in dorsal and ventral view. Scale = 1 mm.

#### Distribution.

China (Guangxi Province: Huaping).

#### Diagnosis.

 The new species resembles *Stenus huanghaoi* Tang & Li, 2008, with which it shares the faint elytral marks, but it may be distinguished by the heterogeneous punctation of the frons (in *Stenus huanghaoi* always similar in size), the shallower pronotal punctation (especially in the median furrow) and smaller body size (in *Stenus huanghaoi* BL: 3.9–4.5 mm).

#### Etymology.

 This species is named in honor of Mr. Zhang Ding-Heng, administrator of the Huaping Nature Reserve, who provided help in various ways during our field work.

#### Biological notes.

All the specimens were collected by sifting the leaves of bamboo and broad-leaved shrubs in a thick forest (Fig. 50).

### Stenus maoershanus

Pan, Tang & Li sp. n.

urn:lsid:zoobank.org:act:7B0F14EB-F7AA-4B85-A751-B2E3A15EEC87

http://species-id.net/wiki/Stenus_maoershanus

Figs 7–8 41–49

#### Type material.

**Holotype:** **CHINA: Guangxi Prov.:** ♂, Xing’an County, Mt. Mao’ershan, alt. 2100 m, 10.VII.2011, TANG Liang & HE Wen-Jia leg. (SHNU). **Paratype:** **CHINA: Guangxi Prov.:** 1♀, same data as holotype. (SHNU).

#### Description.

 BL: 4.3 mm; FL: 2.0 mm.

HW: 0.81–0.82 mm, PW: 0.60–0.61 mm, PL: 0.63–0.64 mm, EW: 0.76 mm, EL:0.71–0.72 mm, SL: 0.54–0.55 mm.

Brachypterous; body brownish black, head darker, anterior margin of labrum, antennae, maxillary palpi and legs yellowish brown, each elytron with a large elongate orange mark near lateral margin, this mark 4/5 as long as and about 3/5 as broad as the respective elytron.

Head 1.07–1.08 times as wide as elytra; interocular area with two broad longitudinal furrows, median portion convex, almost reaching the level of inner eye margins; punctures round, slightly larger and sparser in median area than those near inner margins of eyes, diameter of large punctures as wide as apical cross section of antennal segment II, interstices between punctures smooth, mostly narrower than half the diameter of punctures, those along midline a little wider. Relative length of antennal segments from base to apex 15: 9: 24: 14: 12: 9.5: 9.5: 6.5: 7: 8: 10. Paraglossae oval.

Pronotum 1.04–1.05 times as long as wide, 0.80–0.81 times as wide as elytra; disk with distinct median longitudinal furrow, this furrow about half the length of pronotum; punctation round and confluent, similar to that of head, interstices smooth, much narrower than half the diameter of punctures except for those in median furrow, which may be wider.

Elytra 0.94–0.95 times as long as wide, distinctly constricted at base, lateral margins gently divergent posteriad; punctation similar to that of pronotum, but slightly coarser, interstices similar to those of pronotum.

Legs with hind tarsi 0.74–0.75 times as long as hind tibiae, tarsomere IV strongly bilobed.

Abdomen cylindrical; paratergites very narrow and smooth, present only in abdominal segment III, posterior margin of tergite VII with indistinct palisade fringe; punctation of tergite III–VIII sparse and shallow, gradually becoming finer posteriad, interstices smooth, varying from narrower to much wider than diameter of punctures.

Male. Sternite VII with inconspicuous emargination at middle of posterior margin, anterior to this emargination flattened; sternite VIII (Fig. 41) with semi-circular emargination at middle of posterior margin; sternite IX (Fig. 42) with long apicolateral projections, posterior margin serrate; tergite X (Fig. 43) with posterior margin truncate and slightly emarginated at middle. Aedeagus (Fig. 44) with apical sclerotized portion of median lobe triangular; expulsion hooks absent (probably lost in this specimen); parameres longer than median lobe, each with 8–9 setae on apico-internal margins.

Female. Sternite VIII (Fig. 46) inconspicuously prominent at middle of posterior margin; tergite X (Fig. 47) with posterior margin truncate and slightly emarginated at middle. Valvifers (Fig. 48) each with big apicolateral tooth; spermatheca strongly sclerotized (Fig. 49).

**Figures 7–8.**
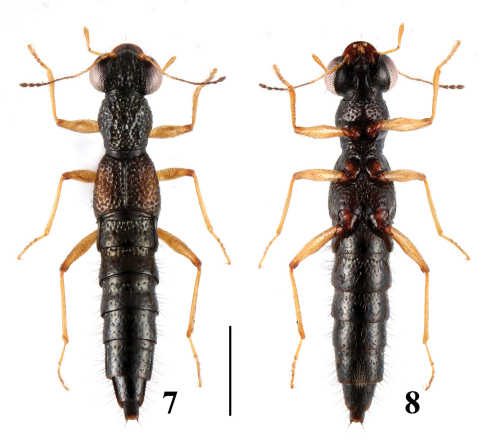
Habitus of*Stenus maoershanus* in dorsal and ventral view. Scale = 1 mm.

#### Distribution.

China (Guangxi Province: Mao’ershan).

#### Diagnosis.

 The new species resembles *Stenus nanlingmotis* Tang & Li, 2008 from Guangdong, but may be distinguished by the broader and deeper pronotal furrow with wider interstices, which may be as wide as diameter of punctures (in *Stenus nanlingmotis* smaller than half the diameter of punctures); the elytral marks are broader, about 3/5 as broad as the respective elytron (in *Stenus nanlingmotis* about 2/5 as broad as the respective elytron).

#### Etymology.

 The specific name is derived from “Mao’ershan”, the type locality of this species.

#### Biological notes.

The female specimen was collected by sifting the leaves of bamboo and broad-leaved shrubs near the mountain summit, the male specimen was collected by beating grass along a drain exposed in sunshine.

### Key to the Chinese species of the Stenus cirrus group

**Table:** 

1	Paraglossae coniform (*flammeus*-complex)	2
–	Paraglossae oval (*cirrus*-complex)	3
2	Body on average broader and larger; punctation of abdominal tergites VI and VII denser, interstices at most as wide as punctures. Habitus: Fig. 5 in Tang et al. 2008; sexual characters: Figs 25–29 in Tang et al. 2008. BL: 3.7–5.7 mm	*Stenus flammeus* Tang & Puthz, 2008 China (Sichuan : Erlangshan)
–	Body on average narrower and slightly smaller; punctation of abdominal tergites VI and VII less dense, interstices up to twice as wide as punctures. Habitus: Fig. 6 in Tang et al. 2008; sexual characters: Figs 30–34 in Tang et al. 2008. BL: 4.0–4.5 mm	*Stenus bostrychus* Tang& Puthz, 2008 China (Sichuan: Hailuogou)
3	Abdominal tergite III without paratergites	4
–	Abdominal tergite III with paratergites	5
4	Elytra unicolored; pronotum and elytra with smooth interstices. Habitus: Figs 1, 2; sexual characters: Figs 9–19. BL: 3.6–4.9 mm	*Stenus fellowesi* Puthz, 2003 China (Hainan: Diaoluoshan)
–	Elytra bicolored; pronotum and elytra with more strongly sculptured interstices. Spermatheca: Fig. 14 in Puthz, 2003; male unknown. BL: 3.0–4.2 mm	*Stenus hainanensis* Puthz, 2003 China (Hainan: Jianfengling)
5	Tergites and sternites of abdominal segments IV–VI separated by sutures	6
–	Tergites and sternites of abdominal segments IV–VI fused without sutures	7
6	Elytra strongly glossy with irregular and slightly confluent punctation, interstices smooth. Sexual characters: Figs 15, 16 in Puthz, 2003. BL: 2.6–3.4 mm	*Stenus huangganmontium* Puthz, 2003 China (Jiangxi: Huanggangshan)
–	Elytra slightly glossy with regular and deep, less distinctly confluent punctation. Sexual characters: Figs 35–39 in Tang et al. 2008. BL: 2.6–3.6 mm	*Stenus cirrus* L. Benick, 1940 China (Zhejiang: Tianmushan)
7	Smaller species, BL ≥ 3.5 mm; elytra unicolored	8
–	Larger species, BL ≤ 3.0 mm; elytra bicolored, with elytral marks or with lateral elytral portion lighter	10
8	Punctation of forebody moderately dense, interstices strongly glossy, punctures defined. Aedeagus: Fig. 26 in Puthz, 2003. BL: 2.5–3.5 mm	*Stenus falsus* L. Benick, 1940 China (Jiangsu: Chinkiang)
–	Punctation of forebody very dense, interstices weakly glossy at most, punctures less defined	9
9	Interstices of pronotal punctation distinctly reticulate. Habitus: Fig. 4 in Tang et al. 2008; sexual characters: Figs 20–24 in Tang et al. 2008. BL: 2.3–2.8 mm	*Stenus shenshanjiai* Tang& Puthz, 2008 China (Zhejiang: Niutoushan)
–	Interstices of pronotal punctation indistinctly reticulate. Habitus: Fig. 2 in Tang, Li and Zhao 2005; sexual characters: Figs 8–11 in Tang, Li and Zhao 2005. BL: 2.3–3.2 mm	*Stenus nigritus* Tang, Li & Zhao, 2005 China (Shaanxi: Qinling)
10	Head narrower than or slightly wider than elytra	11
–	Head distinctly wider than elytra	13
11	Head slightly wider than elytra; elytra wider than long. Aedeagus: Fig. 1 in Puthz, 1983; female unknown. BL: 3.0–3.5 mm	*Stenus splendidulus* Puthz, 1983 China (Guangxi: S. Guilin)
–	Head distinctly narrower than elytra; elytra longer than wide. Two very similar species with fully developed wings	12
12	Aedeagus with narrow apical sclerotized portion, pointed at apex: Fig. 3 in Puthz, 1998. BL: 3.5–4.7 mm	*Stenus guangxiensis* Rougemont, 1984 China (Guangxi, Zhejiang)
–	Aedeagus with broad apical sclerotized portion, rounded at apex: Fig. 4 in Puthz, 1998. BL: 3.3–4,7 mm	*Stenus aeneonitens* Puthz, 1998 China (Sichuan: Qingchengshan)
13	Punctation of frons sparse and heterogeneous	14
–	Punctation of frons dense and almost uniform	15
14	Elytral marks very distinct; punctation of elytra very dense, interstices of basal half of elytra narrow, forming sharp rugae. Male characters: Figs 11, 12 in Puthz, 2003; female unknown. BL: 3.0–4.0 mm	*Stenus cactiventris* Puthz, 2003 China (Guangdong: Dawuling)
–	Elytral marks faint; punctation of elytra less dense, interstices of basal half of elytra relatively broad, not forming sharp rugae. Habitus: Figs 5, 6; sexual characters: Figs 31–40. BL: 3.5–3.9 mm	*Stenus zhangdinghengi* sp. n. China (Guangxi: Huaping)
15	Punctation of pronotum and elytra dense, but not confluent	16
–	Punctation of pronotum and elytra very dense and confluent, that of the pronotum irregular. Species reliably identified only by their sexual characters	19
16	Elytral marks very indistinct; punctation of pronotum homogeneous and mostly well defined. Habitus: Fig. 2 in Tang, Zhao and Li 2008; sexual characters: Figs 8–12 in Tang, Zhao and Li 2008. BL: 3.4–4.0 mm	*Stenus xuwangi* Tang & Li, 2008 China (Guangdong: Nanling)
–	Elytral marks distinct; punctation of pronotum heterogeneous and/or moderately confluent	17
17	Punctation on elytral marks very sparse, interstices partly wider than diameter of punctures. Male characters: Figs 9, 10 in Puthz, 2003; female unknown. BL: 3.2–4.2 mm	*Stenus cooterianus* Puthz, 2003 China (Fujian: Wuyishan)
–	Punctation on elytral marks dense, interstices slightly wider than half the diameter of punctures	18
18	Body larger, BL 3.8–4.5 mm, FL 1.9–2.1 mm; elytral marks longer than half the elytra, extending towards humeral angles. Habitus: Fig. 3 in Tang, Li and Zhao 2005; sexual characters: Figs 12–15 in Tang, Li and Zhao 2005	*Stenus ovalis* Tang, Li & Zhao, 2005 China (Zhejiang: Wuyanling)
–	Body smaller, BL 3.2–4.1 mm, FL 1.6–1.7 mm; elytral marks shorter than half of elytral length, not extending towards humeral angles. Habitus: Fig. 1 in Tang, Li and Zhao 2005; sexual characters: Figs 4–7 in Tang, Li and Zhao 2005	*Stenus andoi* Tang, Li & Zhao, 2005 China (Hubei: Houhe)
19	Punctation of elytra very dense and confluent; male apical emargination of abdominal sternite VIII broad and shallow: Fig. 7 in Tang et al. 2008	20
–	Punctation of elytra less dense, and less confluent; male apical emargination of abdominal sternite VIII narrower, rounded: Fig. 12 in Tang et al. 2008	21
20	Elytral marks shorter, not extending towards humeral angles. Habitus: Fig. 1 in Tang et al. 2008; sexual characters: Figs 7–11 in Tang et al. 2008. BL: 3.7–5.0 mm	*Stenus zhulilongi* Tang & Puthz, 2008 China (Zhejiang: Gutianshan)
–	Elytral marks longer, extending towards humeral angles. Sexual characters: Figs 19, 20 in Puthz, 2003. BL: 3.5–4.7 mm	*Stenus lacrimulus* L. Benick, 1942 China (Fujian: Wuyishan)
21	Elytral marks distinct, less than half the length of elytra; punctation of pronotum less confluent. Habitus: Fig. 3 in Tang et al. 2008; sexual characters: Figs 16–19 in Tang et al. 2008. BL: 3.7–5.0 mm	*Stenus jiulongshanus* Tang & Puthz, 2008 China (Zhejiang: Jiulongshan)
–	Elytral marks more than half the length of elytra (elytral marks of *Stenus huanghaoi* may be ill-defined); punctation of pronotum more confluent	22
22	Median longitudinal pronotal furrow deep, with interstices as wide as diameter of punctures. Habitus: Figs 7, 8; sexual characters: Figs 41–49. BL: 4.3 mm	*Stenus maoershanus* sp. n. China (Guangxi: Mao’ershan)
–	Median longitudinal pronotal furrow shallow or indistinct, with interstices narrower than half the diameter of punctures	23
23	Body larger, FL 2.0–2.2mm	24
–	Body smaller, FL 1.8–1.9mm	25
24	Head relatively wide, HW 0.87–0.96mm, HW/EW 1.12–1.17. Habitus: Fig. 3 in Tang, Zhao and Li 2008; sexual characters: Figs 13–16 in Tang, Zhao and Li 2008. BL: 4.2–4.9 mm	*Stenus nanlingmontis* Tang & Li, 2008 China (Guangdong: Nanling)
–	Head relatively narrow, HW 0.79–0.91mm, HW/EW 1.06–1.12. Habitus: Fig. 2 in Tang et al. 2008; sexual characters: Figs 12–15 in Tang et al. 2008. BL: 3.8–5.0 mm	*Stenus lijinweni* Tang & Puthz, 2008
	China (Jiangxi: Sanqingshan; Zhejiang: Gutianshan)
25	Punctation of pronotum heterogeneous, with moderately coarse to very coarse punctures; elytral marks distinct. Male characters: Figs 17, 18 in Puthz, 2003; female unknown. BL: 3.6–4.3 mm	*Stenus wuyiensis* Puthz, 2003 China (Fujian: Wuyishan)
–	Punctation of pronotum uniform, very coarse; elytral marks indistinct. Habitus: Fig. 1 in Tang, Zhao and Li 2008; sexual characters: Figs 4–7 in Tang, Zhao and Li 2008. BL: 3.9–4.5 mm	*Stenus huanghaoi* Tang & Li, 2008 China (Guangdong: Nanling)

**Figures 9–21.**
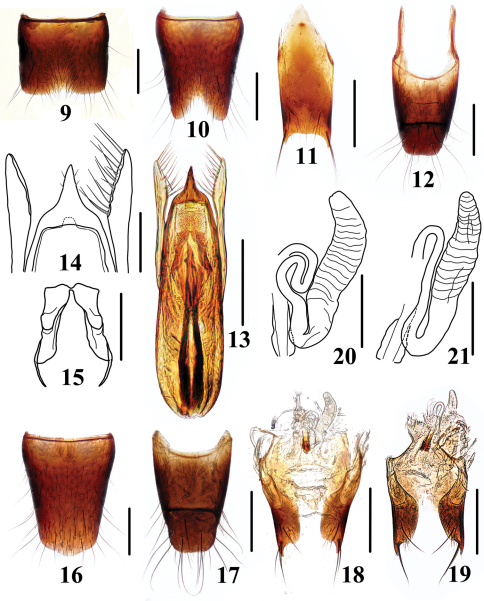
*Stenus fellowesi* **9** male sternite VII **10** male sternite VIII **11** male sternite IX **12** male tergite IX, X **13** aedeagus in ventral view **14** apex of aedeagus **15** expulsion hooks **16** female sternite VIII **17** female tergite IX, X **18–19** valvifers and spermatheca **20–21** spermatheca. Scales = 0.1 mm (14–15, 20–21), scales = 0.25 mm (9–13, 16–19).

**Figures 22–30.**
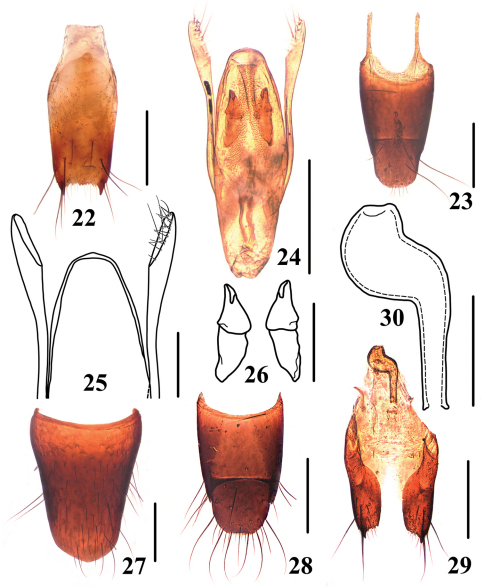
*Stenus huanghaoi* **22** male sternite IX **23** male tergite IX, X **24** aedeagus in ventral view **25** apex of aedeagus **26** expulsion hooks **27** female sternite VIII **28** female tergite IX, X **29** valvifers and spermatheca **30** spermatheca. Scales = 0.1 mm (25–26, 30), scales = 0.25 mm (22–24, 27–29).

**Figures 31–40.**
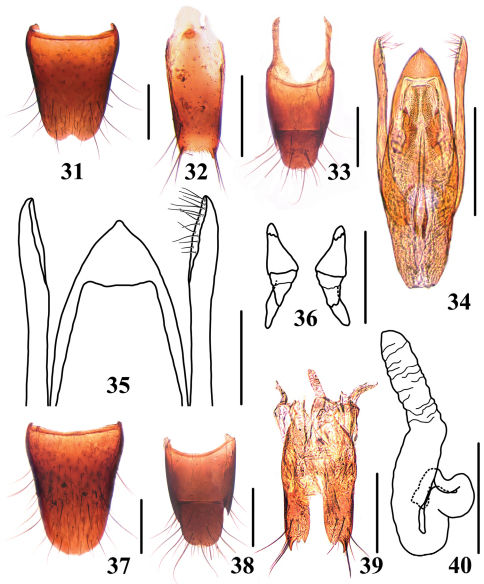
*Stenus zhangdinghengi* **31** male sternite VIII **32** male sternite IX **33** male tergite IX, X **34** aedeagus in ventral view **35** apex of aedeagus **36** expulsion hooks **37** female sternite VIII **38** female tergite IX, X **39** valvifers and spermatheca **40** spermatheca. Scales = 0.1 mm (35–36, 40), scales = 0.25 mm (31–34, 37–39).

**Figures 41–49.**
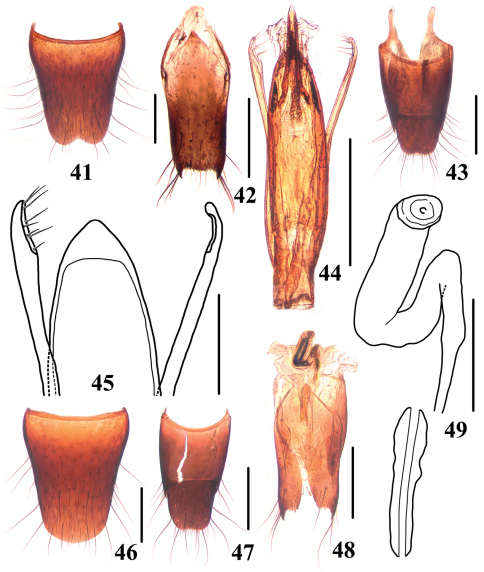
*Stenus maoershanus* **41** male sternite VIII **42** male sternite IX **43** male tergite IX, X **44** aedeagus in ventral view **45** apex of aedeagus **46** female sternite VIII **47** female tergite IX, X **48** valvifers and spermatheca **49** spermatheca. Scales = 0.1 mm (45, 49), scales = 0.25 mm (41–44, 46–48).

**Figure 50.**
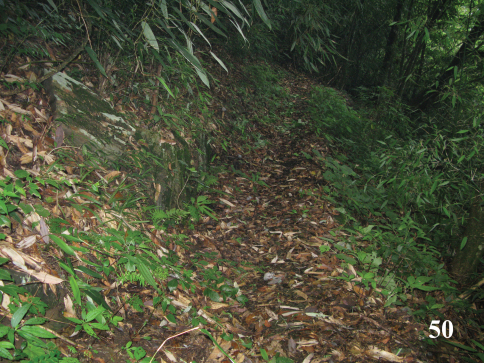
Habitat of *Stenus zhangdinghengi* in Huaping Nature Reserve.

## Supplementary Material

XML Treatment for Stenus fellowesi

XML Treatment for Stenus huanghaoi

XML Treatment for Stenus zhangdinghengi

XML Treatment for Stenus maoershanus
